# XBusNet: Text-Guided Breast Ultrasound Segmentation via Multimodal Vision–Language Learning

**DOI:** 10.3390/diagnostics15222849

**Published:** 2025-11-11

**Authors:** Raja Mallina, Bryar Shareef

**Affiliations:** Department of Computer Science, University of Nevada, Las Vegas, NV 89154, USA

**Keywords:** breast cancer, breast ultrasound, medical image analysis, deep learning, vision transformer (ViT), U-Net, prompt learning, BI-RADS, segmentation, vision–language models (VLMs)

## Abstract

**Background/Objectives:** Precise breast ultrasound (BUS) segmentation supports reliable measurement, quantitative analysis, and downstream classification yet remains difficult for small or low-contrast lesions with fuzzy margins and speckle noise. Text prompts can add clinical context, but directly applying weakly localized text–image cues (e.g., CAM/CLIP-derived signals) tends to produce coarse, blob-like responses that smear boundaries unless additional mechanisms recover fine edges. **Methods:** We propose XBusNet, a novel dual-prompt, dual-branch multimodal model that combines image features with clinically grounded text. A global pathway based on a CLIP Vision Transformer encodes whole-image semantics conditioned on lesion size and location, while a local U-Net pathway emphasizes precise boundaries and is modulated by prompts that describe shape, margin, and Breast Imaging Reporting and Data System (BI-RADS) terms. Prompts are assembled automatically from structured metadata, requiring no manual clicks. We evaluate the model on the Breast Lesions USG (BLU) dataset using five-fold cross-validation. The primary metrics are Dice and Intersection over Union (IoU); we also conduct size-stratified analyses and ablations to assess the roles of the global and local paths and the text-driven modulation. **Results:** XBusNet achieves state-of-the-art performance on BLU, with a mean Dice of 0.8766 and IoU of 0.8150, outperforming six strong baselines. Small lesions show the largest gains, with fewer missed regions and fewer spurious activations. Ablation studies show complementary contributions of global context, local boundary modeling, and prompt-based modulation. **Conclusions:** A dual-prompt, dual-branch multimodal design that merges global semantics with local precision yields accurate BUS segmentation masks and improves robustness for small, low-contrast lesions.

## 1. Introduction

Breast cancer is the most common cancer in U.S. women (about 30% of new cases in 2025), and roughly 42,170 women are expected to die from it [1]. Early detection and timely treatment remain central to lowering mortality [1]. Mammography, magnetic resonance imaging (MRI), and ultrasound are the main imaging modalities used for screening and diagnostic workups [2,3]. Mammography and MRI are accurate, but each has limitations: mammography loses sensitivity in women with dense breast tissue [4], and MRI, though highly sensitive, is costly and impractical for routine use [5]. Ultrasound is safe, painless, nonionizing, affordable, and widely available, which is valuable for younger patients and in settings with limited resources [6]. Its interpretation, however, is highly operator-dependent and is often hindered by speckle noise, heterogeneous tissue appearance, and indistinct lesion boundaries, challenges shown in benchmark datasets such as BUSIS [7,8]. These factors motivate automated segmentation methods that are accurate, robust across imaging conditions, and interpretable to radiologists in routine practice.

Accurate tumor segmentation improves diagnostic precision by enabling measurements aligned with BI-RADS (size, shape, and margins), yielding more reliable radiomics features, keeping classifiers focused on the lesion, and supporting longitudinal tracking [9,10]. Automated segmentation improves reproducibility and can streamline reporting in routine ultrasound. Over the past decade, breast ultrasound (BUS) segmentation has progressed in three broad stages. Early studies used classical image processing—thresholding, edge detection, region growing, and active contours [11]. Next came machine learning pipelines that relied on hand-crafted texture and shape descriptors (e.g., GLCM, local binary patterns, wavelets) paired with supervised classifiers, such as SVMs and random forests, or with clustering and graph-based methods (fuzzy c-means, k-means, conditional random fields, graph cuts) [7]. With deep learning, U-Net and related encoder–decoder models became the standard [12,13]. Variants that add residual or dense connections and attention gates improved boundary quality [14,15,16]. Attention mechanisms and Transformer designs helped capture long-range context [17,18,19], and hybrid CNN–Transformer models reported strong results on BUS data, including a multitask (segmentation and classification) CNN–Transformer trained jointly for both tasks [20,21]. Despite this progress, BUS still presents challenging scenarios: small tumor sizes, low contrast, heterogeneous backgrounds, and variability across scanners and sites. These difficulties are compounded by limited labeled data, class imbalance, and annotation variability. Pixel only training also rarely uses explicit clinical descriptors (e.g., BI-RADS or radiomics), which can make outputs harder to interpret in routine reading [7,22]. Small tumor-aware architectures such as STAN and ESTAN directly target this failure mode [23,24]. In current BUS practice, lesion measurements are predominantly manual; automated, BI-RADS-aligned masks can standardize reporting and reduce inter-operator variability.

To address this gap, prior studies have injected domain knowledge into BUS segmentation in several ways. First, clinical descriptors and radiomics are used as auxiliary supervision or fused with visual features [10,25]. Second, anatomy-aware models and losses encode priors on shape, boundaries, and topology to keep masks plausible [26,27,28,29,30]. Third, ultrasound physics has been leveraged through speckle and attenuation models and quantitative ultrasound features [31,32,33,34]. These strategies improve clinical grounding but still face limits in scalability and generalization, which motivates frameworks that flexibly integrate descriptors while preserving robustness across imaging conditions.

Prompt-based learning treats segmentation as a conditional task steered by auxiliary inputs (“prompts”). In practice, prompts take several forms: (i) Spatial prompts. Image coordinates or regions such as points, bounding boxes, polygons/masks, and scribbles. The Segment Anything Model (SAM) is a canonical spatially promptable segmenter and supports these types at scale [35]. (ii) Textual prompts. Class names or free-form phrases that describe the target. CLIPSeg shows that text or image prompts can condition masks [36]. Pairing open-vocabulary detectors or vision–language models with SAM enables text-conditioned localization and segmentation; for example, pairing Grounding DINO with SAM (“Grounded-SAM”) uses free text to propose regions, and BLIP provides strong image–text pretraining [37,38,39,40,41]. (iii) Other side information. Categorical tags or numeric attributes (e.g., approximate lesion size or laterality) that can be encoded as tokens or embeddings and used alongside visual features.

Adapting these ideas to medical imaging is challenging because lesions often have subtle contrast, boundaries are weak, and domain shift across scanners and sites is common [42]. Several adaptations target these issues: BUSSAM introduces CNN–ViT adapters for breast ultrasound, MedSAM pretrains on large medical datasets, and SAMed applies low-rank adaptation for efficient tuning [43,44,45]. Vision–language approaches have also been explored directly for ultrasound, such as CLIP-TNseg [46]; causal interventions have been proposed to stabilize text guidance under noisy or ambiguous prompts [46,47]. These studies indicate benefits from both spatial and text prompts, yet most methods adopt a single prompt modality. Few approaches jointly exploit global semantic context and fine-grained clinical attributes in a manner consistent with radiologist reasoning. Furthermore, it is desirable that segmentation outputs are consistent with BI-RADS descriptors [9]. Common tools such as Grad-CAM and attention visualizations offer partial insight but often produce diffuse or inconsistent patterns that are difficult to map to clinical terms [48,49,50].

In this work, we present XBusNet, a dual-branch, dual-prompt vision and language framework for BUS segmentation. A global branch based on Contrastive Language Image Pretraining (CLIP) and a Vision Transformer (ViT) is conditioned by a Global Feature Context Prompt (GFCP) that encodes lesion size and centroids to provide scene-level semantics. A local branch based on U-Net with multi-head self-attention preserves boundary detail. The local decoder is modulated by a text conditioned scaling and shifting mechanism, Semantic Feature Adjustment (SFA), which is driven by a Local or Attribute Guided Prompt (LFP) that encodes shape, margin, and Breast Imaging Reporting and Data System (BI-RADS) terms. Prompts are assembled reproducibly from structured metadata, require no manual clicks, and integrate with standard training. We evaluate XBusNet on the BLU dataset using five-fold cross-validation, include ablations to isolate the role of each component, analyze performance across lesion sizes, and provide Grad-CAM visualizations as qualitative views of model focus relative to BI-RADS descriptors. We hypothesize that combining a Global Feature Context Prompt (GFCP; size/location) with a Local Feature Prompt (LFP; shape, margin, BI-RADS) guides the model toward plausible regions while preserving boundaries, improving BUS segmentation—especially for small or low-contrast lesions.

Our contributions are as follows:

- We propose XBusNet, a dual-branch, dual-prompt vision and language segmentation architecture that combines a CLIP ViT global branch conditioned by GFCP with a U-Net local branch with multi-head self-attention that is modulated via SFA driven by LFPs.
- We design a reproducible prompt pipeline that converts structured metadata into natural language prompts, including global prompts for lesion size and centroid and local prompts for shape, margin, and BI-RADS.
- We introduce a lightweight SFA mechanism in the local decoder to inject attribute-aware scaling and shifting, improving boundary focus while preserving fine detail.
- We provide a comprehensive evaluation protocol with five-fold cross-validation, size-stratified analysis, component ablations, and Grad-CAM overlays used as qualitative visualizations of model focus relative to BI-RADS descriptors.

## 2. Materials and Methods

### 2.1. Datasets

We used the Breast Lesions USG (BLU) dataset [51], which contains 256 breast ultrasound scans from 256 patients. Each scan includes expert verified annotations for benign and malignant lesions. Four images with multiple annotated lesions (two tumors in the same image) were excluded because they represent a very small fraction of the dataset (4/256, ≈1.6%), which would yield an imbalanced and hard-to-interpret evaluation for per-image metrics. The remaining 252 images include 154 benign and 98 malignant tumors. This dataset is also referred to as ‘BrEaST’. All images and labels are publicly available and fully de-identified in the BLU (‘BrEaST’) release; no new data were collected and no identifiable information was accessed.

The dataset provides pixel-wise segmentation masks, patient-level clinical attributes such as age, breast tissue composition, signs, and symptoms, image-level BI-RADS labels, and tumor-level diagnostic information such as shape, margin, and histopathology. We considered the BUSI [52] dataset; however, it does not provide the structured clinical text fields (e.g., BI-RADS shape/margin) needed for our prompt construction. Extending XBusNet to cohorts without such fields (via automatic prompt inference) is planned.

### 2.2. Pre-Processing and Prompt Construction

All images were resized to 352×352 pixels (we therefore took H=W=352 throughout). We chose 352 for compatibility with the pretrained ViT/CLIP encoder (patch size, 16; 352=22×16) and to align with the backbone stride (×32). Prompts were derived from structured metadata in the dataset.

The Global Feature Context Prompt encodes lesion size and location. Size is taken from the dataset metadata and discretized using training-set quantiles (small/medium/large). Location handling differs by split: for training images, location is the centroid of the reference segmentation mask computed from image moments,

(1) $$C_{x}=\frac{M_{10}}{M_{00}},\;C_{y}=\frac{M_{01}}{M_{00}},$$

where $M_{00}$ is the mask area and $M_{10}$ and $M_{01}$ are spatial moments. For validation/test images, location is estimated without masks from a first-pass probability map, as detailed in Section 2.7. The Local Feature Prompt encodes clinically used attributes—shape, margin, and BI-RADS—verbalized directly from metadata. Prompts are tokenized and embedded with the CLIP text encoder, as described in the model subsections.

### 2.3. Training Setup

We used five-fold cross-validation. For each fold, we split the 252 annotated images into train and validation sets at an 80 to 20 ratio, ensuring non-overlapping images. Unless stated otherwise, training ran for 1000 iterations per fold with a batch size of 4, the AdamW optimizer, an initial learning rate of $10^{-4}$, and a cosine schedule. Mixed-precision training was enabled with automatic mixed precision.

#### Hyperparameter Tuning and Overfitting Control

Within each cross-validation fold, we performed a small hyperparameter sweep restricted to the fold’s training partition (no images from the fold’s validation/test partitions were used for selection). We varied a compact set of parameters (learning rate, weight decay, batch size, loss weights) while keeping all other settings fixed. The best configuration was chosen by the mean Dice of an internal split of the training partition and then fixed across all folds and methods we implemented for comparability. To mitigate overfitting, we limited the sweep budget, used weight decay (AdamW), and monitored the train–validation gap; we did not use data augmentation or dropout in order to isolate architectural effects. We did not observe unstable divergence across folds. We acknowledge that nested cross-validation would further decouple tuning from evaluation and leave a fully nested protocol to future work.

### 2.4. Evaluation Metrics

We report Dice, Intersection over Union (IoU), the false positive rate (FPR), and the false negative rate (FNR). Let $M\in {\left\{0,1\right\}}^{H\times W}$ be the ground-truth mask, where *H* and *W* are the resized image height and width, and $P\in {\left[0,1\right]}^{H\times W}$ is the predicted probability map. We obtain a binary prediction by thresholding at $\tau_{\mathrm{seg}}=0.5$:

$$\hat{Y}=1\;\left\{\;P\geq \tau_{\mathrm{seg}}\;\right\}\in {\left\{0,1\right\}}^{H\times W}.$$

The pixel-wise counts (sums over all pixels (i,j)) are

$$\mathrm{TP}=\sum_{i,j}{\hat{Y}}_{ij}M_{ij},\;\mathrm{FP}=\sum_{i,j}{\hat{Y}}_{ij}\left(1-M_{ij}\right),\;\mathrm{FN}=\sum_{i,j}\left(1-{\hat{Y}}_{ij}\right)M_{ij},\;\mathrm{TN}=\sum_{i,j}\left(1-{\hat{Y}}_{ij}\right)\left(1-M_{ij}\right).$$

The per-image metrics are

$$\mathrm{Dice}=\frac{2\;\mathrm{TP}}{2\;\mathrm{TP}+\mathrm{FP}+\mathrm{FN}},\;\mathrm{IoU}=\frac{\mathrm{TP}}{\mathrm{TP}+\mathrm{FP}+\mathrm{FN}},$$

$$\mathrm{FNR}=\frac{\mathrm{FN}}{\mathrm{FN}+\mathrm{TP}},\;\mathrm{FPR}=\frac{\mathrm{FP}}{\mathrm{FP}+\mathrm{TN}}.$$

We computed metrics per image and the average within each fold; the fold means were then summarized across the K=5 folds (mean ± SD). For numerical stability, we used $\varepsilon =10^{-8}$ in denominators during implementation.

### 2.5. Implementation Details and Hardware

All experiments were implemented in PyTorch v 2.9.0 and run on a cluster with 8× NVIDIA A100 Tensor Core GPUs (NSF MRI (#2117941)) in SXM form factor. The CLIP encoders were frozen during training. The ResNet50 encoder was initialized from ImageNet weights and fine-tuned. For deployment, we provided an inference-profiling script and notes on memory/throughput; latency depended on hardware and batch size.

### 2.6. Overall Architecture

**Figure 1:**
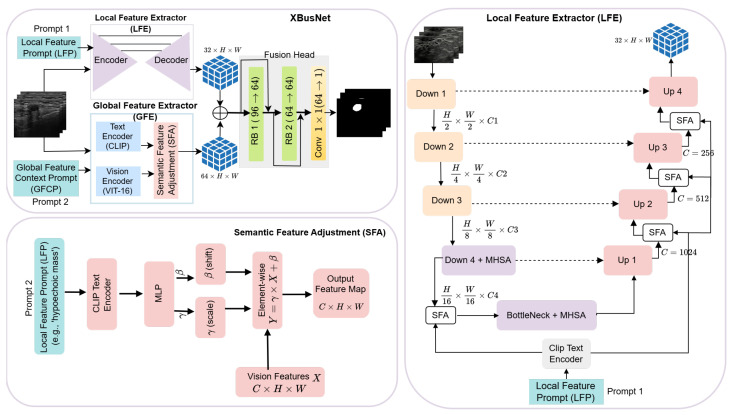
Overview of the proposed XBusNet architecture, showing the Local Feature Extractor (LFE), Global Feature Extractor (GFE), and Semantic Feature Adjustment (SFA) modules. RB = residual block; MHSA = multi-head self-attention.

XBusNet is a text-guided medical image segmentation framework that integrates fine-grained local context from medical images with global semantic cues derived from natural language prompts. The model follows a dual-branch, dual-prompt vision and language design with a Global Feature Extractor (GFE) and a Local Feature Extractor (LFE) that run in parallel and exchange information through a Semantic Feature Adjustment (SFA) mechanism (see Figure 1). The GFE uses a CLIP-based Vision Transformer with a patch size of 16 to capture global semantic relationships conditioned on a high-level conditional prompt, $p_{c}$. The LFE uses a ResNet50 encoder with Transformer blocks at deep levels and a U-Net-style decoder to represent spatial detail under a phrase-level prompt, $p_{\ell }$.

Let $I\in R^{B\times 3\times H\times W}$ denote the input image batch and let $p_{c}$ and $p_{\ell }$ be the conditional and local prompts. (Here, *H* and *W* denote the resized input-image size, whereas *h* and *w* denote the feature-map size at the working resolution.)

The two branches produce

(2) $$F_{g}=\mathrm{GFE}\left(I,p_{c}\right),\;F_{\ell }=\mathrm{LFE}\left(I,p_{\ell }\right),$$

with $F_{g}\in R^{B\times C_{g}\times h\times w}$ and $F_{\ell }\in R^{B\times C_{\ell }\times h\times w}$. (Here, $C_{g}$ and $C_{\ell }$ denote the channel widths; in our implementation, $C_{g}=64$ and $C_{\ell }=32$). SFA modulates features in both branches using scaling and shifting parameters computed from the corresponding prompt embedding:

(3) $$\begin{array}{cccc} {\hat{F}}_{g} & =\gamma_{g}⊙F_{g}+\beta_{g},\; & \;[\gamma_{g},\beta_{g}] & =\mathrm{SFA}(F_{g},e_{c}),\\ {\hat{F}}_{\ell } & =\gamma_{\ell }⊙F_{\ell }+\beta_{\ell },\; & \;[\gamma_{\ell },\beta_{\ell }] & =\mathrm{SFA}(F_{\ell },e_{\ell }), \end{array}$$

where ⊙ denotes element-wise multiplication. For channel-wise FiLM, $\gamma_{g},\beta_{g}\;\in \;R^{B\times C_{g}\times 1\times 1}$ and $\gamma_{\ell },\beta_{\ell }\;\in \;R^{B\times C_{\ell }\times 1\times 1}$, broadcast over (h,w) (not pixel-wise). Prompt embeddings $e_{c},e_{\ell }\in R^{B\times e}$ (with *e* being the text-embedding dimension) are linearly projected to a fixed width, *d*, for conditioning; we use the same fixed *d* throughout, including Equation (9). A residual form can be used at implementation time as $F_{\mathrm{out}}=\hat{F}+F$.

The fusion head integrates the modulated features and prepares them for prediction:

(4) $$F_{\mathrm{fused}}=\Phi_{\mathrm{fuse}}\;\left([{\hat{F}}_{g},{\hat{F}}_{\ell }]\right),$$

Here, $\Phi_{\mathrm{fuse}}$ denotes the two residual blocks described in Section 2.10. This is followed by a 1×1 convolution that yields the segmentation logits:

(5) $$\hat{Y}={\mathrm{Conv}}_{1\times 1}\left(F_{\mathrm{fused}}\right),\;\hat{Y}\in R^{B\times 1\times H\times W}.$$

This organization lets the network use language to steer scene-level context while the local pathway preserves boundaries and fine structure.

### 2.7. Global Feature Extractor (GFE)

#### 2.7.1. Global Features and Prompts

Global features comprise size and location. Lesion size is read from the dataset metadata and discretized using training-set quantiles (small/medium/large). Location handling differs by split: (i) Training. the location is the centroid $\left(C_{x},C_{y}\right)$ of the reference segmentation mask, computed from the image moments $C_{x}=M_{10}/M_{00}$, and $C_{y}=M_{01}/M_{00}$. (ii) Validation/Test. The location is estimated from a first-pass probability map, *P*, by running the model without spatial text, thresholding at τ=0.30, retaining the largest connected component to form a coarse proposal, *R*, and computing its centroid, $\left({\hat{C}}_{x},{\hat{C}}_{y}\right)$. Centroids are mapped to breast quadrants (upper/lower × inner/outer) to produce a location token. Size and location tokens are verbalized to form the Global Feature Context Prompt $p_{c}$. Quantiles and thresholds are fixed a priori per fold using only the corresponding training split.

#### 2.7.2. GFE Computation

The GFE computes a scene-level representation conditioned on $p_{c}$ using a Vision Transformer. The prompt is embedded by the (frozen) text encoder to obtain $e_{c}\in R^{B\times e}$ (with *e* being the text-embedding dimension).

Selected Transformer layers provide token sequences, ${\left\{Z^{\left(j\right)}\right\}}_{j\in L}$, with $Z^{\left(j\right)}\in R^{B\times N\times D}$. Here, L=|L| is the number of selected layers, *N* the token count, and *D* the hidden token size. Tokens are linearly reduced to a common width and aggregated:

(6) $$A=\sum_{j\in L}W^{\left(j\right)}\;Z_{\left[:,\;1:\;,\;:\right]}^{\left(j\right)}\;\in \;R^{B\times (N-1)\times r},$$

where the class token is removed. Here *r* is the reduced width, and j∈L indexes the selected layers with weights, $W^{\left(j\right)}$. When attention logits, *A*, are used, we write $\tilde{A}=\mathrm{softmax}\left(A\right)$. Prompt conditioning is applied as channel-wise scaling and shifting of the reduced tokens at a chosen Transformer depth:

(7) $$c=W_{c}\;e_{c},\;\tilde{A}=\left(W_{\mathrm{mul}}c\right)⊙A+\left(W_{\mathrm{add}}c\right).$$

Here *c* denotes the channel count of the reduced tokens; $W_{\mathrm{mul}}$ and $W_{\mathrm{add}}$ are learned linear maps that yield multiplicative/additive FiLM terms. The conditioned tokens are reshaped to a spatial grid and projected with a transposed convolution to obtain the global feature map

(8) $$F_{g}\in R^{B\times 64\times h\times w}.$$

SFA in the global branch follows the affine form in Equation (13) and produces ${\hat{F}}_{g}$ as in Equation (3). The vision and text encoders are frozen; only the reduction, conditioning, and projection layers are trained.

### 2.8. Local Feature Extractor (LFE)

#### 2.8.1. Local Features and Prompts

Local features comprise shape, margin, and *BI-RADS*. These attributes are read from the dataset metadata and verbalized to form the Local Feature Prompt $p_{\ell }$ (e.g., “irregular shape, microlobulated margin, BI-RADS 4”). The mapping from metadata to tokens is deterministic and applied identically across folds.

#### 2.8.2. LFE Computation

The LFE recovers boundaries and fine structure under guidance from $p_{\ell }$. We use a ResNet50 encoder with Transformer blocks at deep levels and a U-Net-style decoder. The prompt is embedded with the text encoder,

(9) $$e_{\ell }=T\left(p_{\ell }\right)\in R^{B\times e},$$

and is linearly projected to the same fixed width, *d*, for SFA in the specified stages. Note: T(·) denotes the (frozen) text encoder. Given $I\in R^{B\times 3\times H\times W}$, the encoder yields the features {enc1,enc2,enc3,enc4,enc5}. We add a Transformer block in the fourth encoder stage and at the bottleneck:

(10) $$\mathrm{enc}4^{\prime }=\mathrm{TrEnc}\left(\mathrm{enc}4\right),\;{\mathrm{center}}^{\prime }=\mathrm{TrEnc}\left(\mathrm{enc}5\right).$$

The decoder upsamples and merges skip connections using up blocks to produce dec4,dec3,dec2,dec1, followed by a final up projection to obtain a 32-channel map at the decoder output.

Semantic Feature Adjustment (SFA) uses the local prompt embedding in encoder stage four and after the first three decoder up blocks:

(11) $$\begin{array}{cc} \mathrm{enc}4^{⋆} & =\mathrm{SFA}(\mathrm{enc}4^{\prime },\;e_{\ell }),\\ \mathrm{dec}4^{⋆} & =\mathrm{SFA}(\mathrm{dec}4,\;e_{\ell }),\\ \mathrm{dec}3^{⋆} & =\mathrm{SFA}(\mathrm{dec}3,\;e_{\ell }),\\ \mathrm{dec}2^{⋆} & =\mathrm{SFA}(\mathrm{dec}2,\;e_{\ell }). \end{array}$$

The final local representation is taken after the last decoder stage:

(12) $$F_{\ell }\in R^{B\times 32\times h\times w}.$$

This configuration preserves boundary detail and small structures while injecting attribute cues in the stages listed above. Unless stated otherwise, the text encoder is frozen, and the LFE encoder–decoder parameters are trainable.

### 2.9. Semantic Feature Adjustment (SFA)

SFA injects prompt driven semantics into feature maps through channel-wise scaling and shifting. The module takes a visual tensor and a prompt embedding, predicts modulation parameters, and applies an affine transformation with an optional residual (see Figure 1).

Let $F\in R^{B\times C\times h\times w}$ be a feature map and let $e\in R^{d}$ be the embedding of the associated prompt. The core operation is

(13) $$\begin{array}{cc} \hat{F} & =\gamma ⊙F+\beta ,\\ \left[\gamma ,\;\beta \right] & =\Psi (e), \end{array}$$

where ⊙ denotes element-wise multiplication and $\gamma ,\beta \in R^{B\times C\times 1\times 1}$ broadcast over spatial dimensions. Here Ψ denotes the per-stage projection network (two-layer MLP) that maps the prompt embedding to (γ,β). Parameters are produced by a small projection network for each stage, *k*, with channel width, $C^{\left(k\right)}$, and are broadcast over spatial dimensions:

(14) $$\begin{array}{cc} z^{\left(k\right)} & ={\mathrm{MLP}}^{\left(k\right)}\left(e\right)\in R^{2C^{\left(k\right)}},\\ \left[\gamma^{\left(k\right)},\;\beta^{\left(k\right)}\right] & =\mathrm{split}\;\left(z^{\left(k\right)}\right), \end{array}$$

Thus, $\gamma^{\left(k\right)},\beta^{\left(k\right)}\in R^{B\times C^{\left(k\right)}\times 1\times 1}$ are per-sample, per-channel FiLM parameters broadcast over (h,w).

In the global branch, we condition on $e_{c}$ to obtain ${\hat{F}}_{g}$; in the local decoder, we condition on $e_{\ell }$ to obtain ${\hat{F}}_{\ell }$, as summarized in Equation (3).

An additive skip may be used in implementation:

(15) $$F_{\mathrm{out}}=\hat{F}+F.$$

### 2.10. Feature Fusion and Prediction Head

We concatenate the modulated global and local feature maps to obtain

(16) $$F_{\mathrm{cat}}={\hat{F}}_{g}\;∥\;{\hat{F}}_{\ell }\;\in \;R^{B\times 96\times h\times w},$$

and refine with two residual blocks followed by a 1×1 convolution:

(17) $$F_{1}={\mathrm{RB}}_{1}\left(F_{\mathrm{cat}}\right),\;F_{\mathrm{fused}}={\mathrm{RB}}_{2}\left(F_{1}\right),\;\hat{Y}={\mathrm{Conv}}_{1\times 1}\left(F_{\mathrm{fused}}\right).$$

## 3. Results

We followed the training setup in Section 2.3 and the metrics in Section 2.4. All experiments used the implementation and hardware in Section 2.5.

### 3.1. Overall Performance

We compared XBusNet with representative non-prompt baselines including U-Net [12], U-ResNet, U_KAN [53], and AnatoSegNet [54], as well as prompt-guided baselines such as BUSSAM [43] and CLIP_TNseg [46]. All models were evaluated on BLU under the same five-fold cross-validation protocol (Section 2.3), the same pre-processing (Section 2.2), and a common operating threshold, $\tau_{\mathrm{seg}}=0.5$ (Section 2.4). We report the mean ± SD across folds for Dice, IoU, the *FPR*, and the *FNR* in Table 1 and provide a size-stratified analysis in Table 2.

#### 3.1.1. Fold-Wise Performance

With five-fold cross-validation (Table 3), XBusNet attained consistently strong overlap across folds (Dice: 0.8583–0.8910; IoU: 0.7987–0.8302) with a stable FPR and FNR, indicating reliable behavior across splits.

#### 3.1.2. Comparison with Prior Methods

Table 1 summarizes mean cross-validation performance against U-Net variants, anatomy-aware models, and text-guided segmenters. XBusNet achieved the best Dice (0.8766) and IoU (0.8150) and the lowest FNR (0.0775), reflecting fewer missed lesion pixels. The FPR is comparable to that of the text-guided baseline and higher than that of some convolutional baselines that tend to under-segment; this trade-off favors recovering lesion extent while keeping spurious activations controlled.

#### 3.1.3. Qualitative Assessment

**Figure 2:**
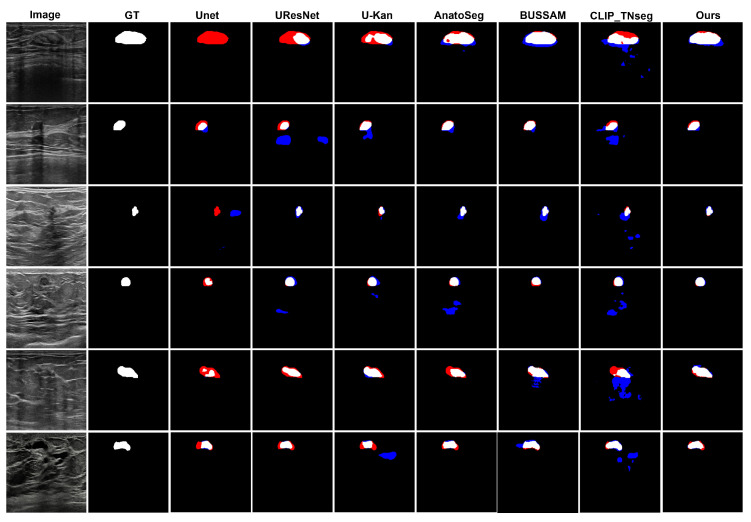
Qualitative comparison of breast ultrasound segmentations. False positives (FPs) are shown in blue and false negatives (FNs) in red.

**Figure 3:**
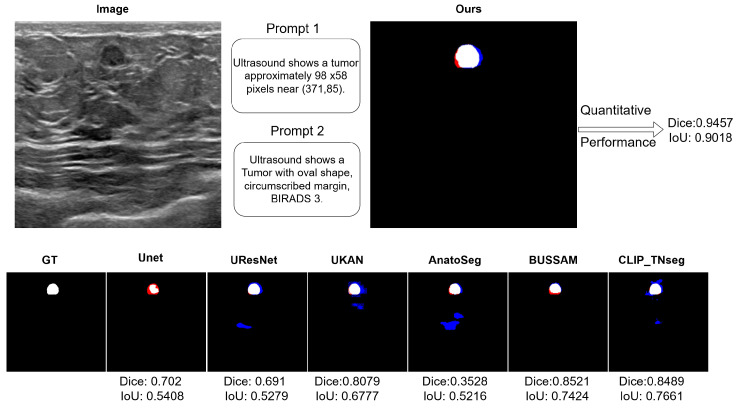
A qualitative example with the exact prompts used for a case. Top left: An input breast ultrasound (BUS) image showing the Global Feature Context Prompt (GFCP) and the Local Feature Prompt (LFP). Top right: Qualitative and quantitative results for XBusNet in this case. Bottom row: Segmentation overlays; ground truth is in white, false negatives are in red, and false positives are in blue.

Figure 2 shows typical error modes with false positives (blue) and false negatives (red). First, for high-contrast masses, U-Net variants exhibited small gaps at the posterior margin (missed pixels), whereas XBusNet produced continuous contours with fewer misses, consistent with the lower FNR in Table 1. Second, on small or faint lesions, several baselines fragmented the mask or missed the target entirely; XBusNet recovered a larger fraction of the lesion area, mirroring the gains in the 0–110 px bin in Table 2. Third, in low-contrast heterogeneous backgrounds, some text-guided baselines could add foreground in adjacent tissue, increasing the FPR; XBusNet suppressed these spurious activations while retaining the faint rim. Finally, for irregular echotexture, convolutional baselines scattered foreground outside the mass, while XBusNet better followed the irregular edge. Figure 3 shows an example of the exact prompts used in the proposed work.

#### 3.1.4. Statistical Analysis and Comparison

All between-method comparisons were performed using image-level Dice and IoU using paired Wilcoxon signed-rank tests; when testing XBusNet against multiple baselines, *p*-values were adjusted via Benjamini–Hochberg FDR (α=0.05). Uncertainty was summarized with 95% bootstrap confidence intervals (10,000 image-level resamples) and is reported in Table 1 and Table 2. Under this protocol, XBusNet significantly outperformed all baselines (adjusted two-sided $p<10^{-4}$). Effect sizes (rank-biserial correlation) indicated large advantages (median $|r_{rb}|\approx 0.82$ for Dice; median $|r_{rb}|\approx 0.80$ for IoU).

### 3.2. Ablation Study

We quantified the contribution of the Local Feature Extractor (LFE), the Global Feature Extractor (GFE), and Semantic Feature Adjustment (SFA) by toggling each component. Table 4 shows that removing any single component degraded performance. Without the LFE, Dice fell from 0.8766 to 0.8572 and IoU from 0.8150 to 0.7865, underscoring the role of local detail and boundary focus. Without the GFE, Dice dropped to 0.8453 and IoU to 0.7772, highlighting the value of global semantic cues from the conditional prompt. Without SFA, Dice decreased to 0.8600 and IoU to 0.8068, showing the benefit of prompt-conditioned modulation that aligns visual features with clinical attributes. The full model with all three components yielded the best results, indicating complementary effects.

### 3.3. Qualitative Visualizations

**Figure 4:**
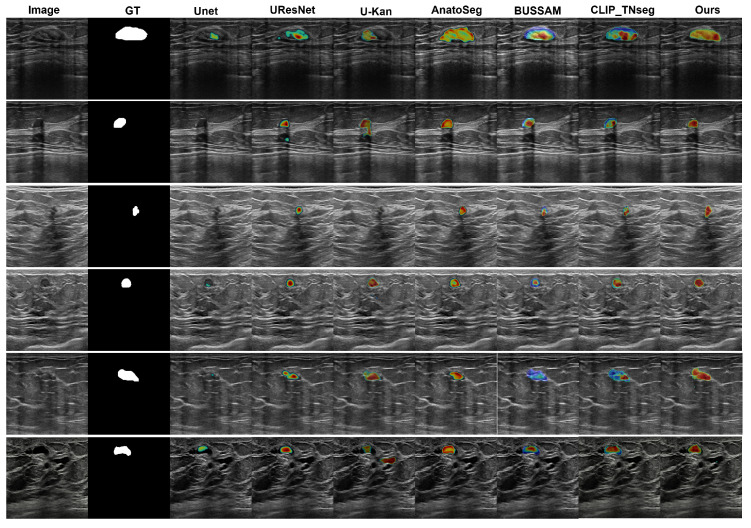
Grad-CAM comparison. Colors map low→high attribution (blue→red), normalized to [0, 1] with a shared scale across images/models.

We employed Grad-CAM overlays to visualize image regions that most influenced the predicted masks (Figure 4). Among the compared methods, U-Net [12], U-ResNet, U-KAN [53], and AnatoSegNet [54] are non-prompt methods; BUSSAM [43] and CLIP-TNseg [46] are prompt-guided; and XBusNet uses dual prompts (global and attribute-conditioned). Non-prompt baselines frequently display diffuse responses in low-contrast tissue, particularly posterior to the lesion, corresponding to missed or fragmented masks. Prompt-guided methods improve spatial correspondence; CLIP-TNseg may still exhibit scattered hotspots around speckle, whereas XBusNet shows concentrated activation adjacent to lesion boundaries and within the mass. For very small lesions, the salient regions for XBusNet overlap the recovered mask more consistently, in line with the small-lesion gains reported in Table 2. These visualizations are qualitative and are not treated as formal explanations.

## 4. Discussion

XBusNet showed strong performance on BLU. Across five folds, the model achieved a mean Dice of 0.8766 and an IoU of 0.8150, with narrow fold-to-fold variation, which suggests stable behavior under fold-wise splits. Compared with U-Net variants, anatomy-aware models, and text-guided baselines, XBusNet also produced a lower false negative rate, indicating fewer missed lesion pixels. The false positive rate was comparable to that of text-guided baselines but higher than that of some convolutional models that under-segment; this trade-off favors recovering lesion extent and can be tuned by adjusting the operating threshold when a stricter specificity is required. Findings align with prior BUS segmentation work where prompt signals improve localization under low contrast; remaining limits include single-cohort evaluation and reliance on structured text fields.

Size-stratified analysis supports these observations. The largest relative gains appear for the smallest lesions, where the model maintains competitive Dice and IoU, while performance remains strong for medium and large lesions. This pattern is consistent with the design: global prompt cues (size and approximate location) guide the search toward plausible regions, and local attribute cues (shape, margin, BI-RADS) help the decoder maintain sharp boundaries when pixel evidence is weak.

Ablation results point to complementary roles for both branches and for Semantic Feature Adjustment. Removing the local branch reduces Dice and IoU, highlighting the importance of boundary detail. Disabling the global branch leads to a larger drop, showing that scene-level cues improve localization and reduce misses. Turning off Semantic Feature Adjustment also degrades performance, which indicates that prompt-conditioned scaling and shifting adds value beyond the two-branch backbone. Together, these findings support the view that the full configuration is needed to realize the observed gains.

Qualitative panels mirror these trends. On high-contrast masses, several baselines show small gaps at the posterior margin, whereas XBusNet yields continuous contours, in line with the lower false negative rate. For small or faint lesions, baselines may fragment or miss the target, while XBusNet recovers a larger fraction of the lesion area. In heterogeneous backgrounds, some text-guided baselines spill into adjacent tissue; XBusNet restrains these activations while preserving the rim. With irregular echotexture, convolutional baselines scatter foreground outside the mass, whereas XBusNet follows the irregular edge more closely. Grad-CAM overlays are used as qualitative views of model focus and are not treated as formal explanations.

### Clinical Relevance

There is no established minimal clinically important difference (MCID) for segmentation metrics in breast ultrasound. Nevertheless, our error profile—particularly improved coverage on small lesions and smoother boundaries—suggests potential benefits for measurement stability (e.g., size/margins aligned with BI-RADS), reduced inter-reader variability on follow-up, and more reliable pre-read triage. Future reader studies should translate Dice/IoU gains into task-level endpoints such as boundary error (mm), read time, and inter-reader variance to anchor a practical MCID. This study is limited by the availability of suitable public data. To our knowledge, there are no widely available breast ultrasound datasets that jointly provide pixel-level masks and rich clinical descriptors such as BI-RADS terms, structured lesion metadata (e.g., shape and margin), and histopathological outcomes. This is the main reason we did not combine BLU with a public external cohort for evaluation. As a practical recommendation for future dataset releases, we encourage curators to include not only classification labels and segmentation masks but also standardized BI-RADS descriptors, acquisition metadata, radiology reports, and, when possible, histopathology results. Such completeness would enable rigorous text-guided segmentation research, cross-dataset evaluation, and clinically meaningful analyses. While structured BI-RADS fields facilitate prompting, entries can be missing or partial; ablations indicate graceful degradation without text cues, and future work will explore automatic prompt inference from images or reports. In practice, the local/global cues can be extracted from routine structured reporting (BI-RADS, shape, margin, size/quadrant). When such fields are absent, XBusNet remains functional with neutral prompts or image-only input; ablations indicate that Dice decreases from 0.8766 to 0.8453 without the GFE (no global prompt) and to 0.8600 without SFA (no prompt-driven modulation). As future work, we will address partially missing fields via automatic prompt inference (image/report-driven) and investigate radiomics-derived surrogates or pseudo-labels.

## 5. Conclusions

XBusNet fuses global prompts (size/location) with local prompts (shape, margin, BI-RADS) and applies Semantic Feature Adjustment to guide segmentation. On BLU, it achieves state-of-the-art Dice/IoU with the largest gains on small lesions, reducing missed pixels while keeping spurious activations controlled. Ablations show that the global branch, the local branch, and the prompt-conditioned modulation each contribute; together they yield balanced boundary quality and region coverage. These results indicate that simple, automatically assembled text cues can strengthen ultrasound segmentation without changes to current imaging practice. Integration options include (i) pre-read triage to flag small/low-contrast candidates and (ii) post-read standardization of lesion measurements; prompts can be auto-assembled from routine fields with minimal workflow impact.

### Future Work

We will test cross-center generalization, add boundary-oriented metrics and calibration analysis, and explore coupling with detection and structured reporting.

## Figures and Tables

**Table 1:** Overall comparison on BLU (five-fold CV). Dice/IoU cells show the fold mean ± SD at a fixed threshold, $\tau_{\mathrm{seg}}=0.5$, with 95% image-level bootstrap CIs in brackets. Lower is better for the FPR and FNR.

Method	Dice	IoU	FPR	FNR
U-Net [12]	0.604 ± 0.038 [0.564, 0.643]	0.500 ± 0.032 [0.463, 0.537]	0.019 ± 0.015	0.342 ± 0.069
U-ResNet	0.721 ± 0.040 [0.687, 0.754]	0.621 ± 0.049 [0.588, 0.654]	0.010 ± 0.007	0.264 ± 0.078
U-KAN [53]	0.752 ± 0.028 [0.739, 0.766]	0.614 ± 0.037 [0.599, 0.630]	0.009 ± 0.001	0.305 ± 0.036
AnatoSegNet [54]	0.733 ± 0.072 [0.702, 0.763]	0.627 ± 0.084 [0.596, 0.658]	0.015 ± 0.013	0.216 ± 0.034
BUSSAM [43]	0.833 ± 0.020 [0.815, 0.850]	0.735 ± 0.026 [0.713, 0.755]	0.014 ± 0.003	0.101 ± 0.024
CLIP-TNseg [46]	0.839 ± 0.019 [0.826, 0.852]	0.765 ± 0.020 [0.750, 0.778]	0.189 ± 0.002	0.134 ± 0.017
XBusNet (Ours)	0.876 ± 0.014 [0.863, 0.889]	0.815 ± 0.013 [0.800, 0.829]	0.099 ± 0.002	0.078 ± 0.012

**Table 2:** Dice and IoU scores (means with 95% CIs) across tumor-length intervals. Bold indicates the best per column.

Model/Length	0–110		111–250		250+	
	Dice	IoU	Dice	IoU	Dice	IoU
U-Net [12]	0.3668	0.2750	0.6461	0.5251	0.6739	0.5625
	[0.3211, 0.5105]	[0.2467, 0.4135]	[0.5992, 0.6948]	[0.4928, 0.5843]	[0.5776, 0.7310]	[0.4779, 0.6246]
U-ResNet	0.6238	0.5327	0.7859	0.6863	0.7305	0.6217
	[0.5364, 0.7157]	[0.4451, 0.6092]	[0.7371, 0.8129]	[0.6385, 0.7153]	[0.5932, 0.7400]	[0.4885, 0.6393]
U-Kan [53]	0.7256	0.5817	0.7584	0.6216	0.7312	0.5892
	[0.7052, 0.7653]	[0.5574, 0.6274]	[0.7442, 0.7786]	[0.6049, 0.6457]	[0.7117, 0.7723]	[0.5685, 0.6398]
AnatoSegNet [54]	0.6303	0.5215	0.7843	0.6822	0.7207	0.6131
	[0.5375, 0.7161]	[0.4425, 0.6097]	[0.7411, 0.8056]	[0.6293, 0.7026]	[0.6539, 0.7841]	[0.5492, 0.6807]
BUSSAM [43]	0.7846	0.6714	0.8465	0.7510	0.8426	0.7495
	[0.7364, 0.8271]	[0.6171, 0.7205]	[0.8255, 0.8659]	[0.7241, 0.7762]	[0.8034, 0.8765]	[0.7026, 0.7911]
CLIP-TNseg [46]	0.7689	0.7026	0.8587	0.7867	0.8447	0.7621
	[0.7375, 0.8133]	[0.6725, 0.7450]	[0.8462, 0.8750]	[0.7732, 0.8057]	[0.8169, 0.8605]	[0.7255, 0.7777]
XBusNet (Ours)	0.8507	0.7925	0.8947	0.8388	0.8553	0.7774
	[0.8136, 0.8852]	[0.7545, 0.8280]	[0.8776, 0.9098]	[0.8204, 0.8561]	[0.8291, 0.8796]	[0.7469, 0.8069]

**Table 3:** Fold-wise validation performance of XBusNet (latest run).

Fold	Dice	IoU	FPR	FNR
0	0.8846	0.8241	0.0984	0.0670
1	0.8910	0.8302	0.1280	0.0659
2	0.8583	0.7987	0.1077	0.0835
3	0.8649	0.8033	0.0857	0.0771
4	0.8836	0.8181	0.0771	0.0944
Mean	0.8766	0.8150	0.0994	0.0776

**Table 4:** Ablation study on effect of LFE, GFE, and SFA modules. The bold indicates the best per column.

LFE	GFE	SFA	Dice	IoU
Yes	Yes	Yes	0.8766	0.8150
No	Yes	No	0.8572	0.7865
Yes	No	Yes	0.8453	0.7772
Yes	Yes	No	0.8600	0.8068

## Data Availability

Training and inference code, configurations, and evaluation scripts are available at github.com/AAR-UNLV/XBusNet, and the dataset is accessed on 9 May 2025 through https://www.cancerimagingarchive.net/collection/breast-lesions-usg/.
